# The Impact of Climate-Smart Agriculture Technology on Productivity: Does Row Planting Matter? Evidence from Southern Ethiopia

**DOI:** 10.1155/2022/3218287

**Published:** 2022-08-12

**Authors:** Workineh Ayenew Mossie

**Affiliations:** Debre Berhan University, Debre Berhan, Ethiopia

## Abstract

The impact of climate-friendly agricultural practices on rural household productivity is not well understood, and the available evidence is mainly qualitative. Therefore, this study investigated the impact of the introduction of Climate-Smart Agriculture Practices (CSA, i.e., row planting) on the productivity of improved wheat producers of rural farmer households in Misha Woreda, the southern region of Ethiopia. For this study, we used the data collected from 202 randomly selected wheat producers through a structured questionnaire. The data were analyzed using propensity score matching (PSM) and the generalized Roy model of the semiparametric local instrument variable (LIV) method. The results of the PSM estimation showed that wheat row planting has a positive and significant impact on productivity. The study found that farmers who sowed wheat in a row produced 1368 kg of wheat per hectare compared to the counterfactual scenario. To further validate whether this result is a pure effect of the row planting technique, we performed a covariate balance test that confirmed the insensitivity of the treatment effect estimates to unobserved selection bias. In addition, the Marginal Treatment Effect (MTE) model also showed that the marginal utility of row planting adoption increases the propensity of farmers to adapt climate-smart agriculture technologies. Therefore, by increasing the productivity of farm households, the expansion of technology will significantly contribute to farmers' resilience to the harmful effects of climate change and welfare.

## 1. Introduction

Climate change is becoming a growing challenge to maintaining global food security that primarily affects the livelihoods of rural households in developing countries [1–3]. Maintaining food security and improving the livelihoods of rural households in Ethiopia is a major challenge resulting from rapid population growth, recurring droughts, land degradation, increasing rural-urban migration, low agricultural production, decreasing land area per capita, and backward agricultural technology [2, 4]. According to the World Food program [4], 31 per cent of households in Ethiopia are calorie-deficient (<2550 kcal per adult, equivalent per day), with high levels of insecurity in rural areas (33 per cent of households) high. The Ethiopian economy depends on agriculture for the generation of jobs, food, sources of foreign exchange, and supplies for the local industry. Despite this significant reliance on the sector, agriculture lags behind, meaning it is rain-fed, and farmers are smallholders with traditional farming tools. According to Timothy et al. [3], climate change and variability will result in an enormous loss of crop production in the coming years. Therefore, the development of climate resilient agriculture plays an outstanding role in the livelihood of the rural poor and the population as a whole.

Since the introduction of the Climate Resilient Green Economy (CRGE) strategy in 2011, Ethiopia has built up climate-friendly agriculture. The design aims primarily to increase food security and improve farm income by introducing improved farming practices and infrastructure while protecting the quality of the environment. Climate-smart agricultural practices (CSA) are mainly yield-enhancing and reduce the challenges of climate variability [5]. Adopting these high yield technologies will help build a climate resilient economy by adapting to shocks such as drought and disease, which will ultimately increase agricultural productivity, improve food security, and reduce poverty. Numerous studies have shown the positive effects of CSA on livelihoods. Studies by Tolosa et al. [6], Abate et al. [7], and Mengie et al. [8] found that CSA has a positive impact on increasing crop yield in Ethiopia; Teklewold et al. [9], Fentie and Beyene [5] in Ethiopia, and Martey et al. [10] in Ghana found positive effects on improving welfare (farm income and per capita consumption per adult equivalent). Teklewold et al. [9] found positive effects of CSA on multidimensional poverty reduction in Ethiopia. Mujeyi et al. [11] also argued that food security and farmers' income respond positively to climate-smart agriculture in Zimbabwe. Despite climate-friendly agriculture (CSA) practices making such positive contributions to improving the welfare of rural smallholder households, there are few studies on the introduction and impact of such practices on agricultural productivity. In addition, most studies that focus on climate-smart farming practices for well-being overlooked the role of row planting, particularly on wheat productivity (the exception is [5] on teff).

Many agricultural technologies, such as fertilizers, improved varieties, and water management practices, have immense potential for increasing yields. However, they are capital intensive, less affordable, and therefore their adoption rate is lower. Thus, despite efforts to increase the uptake of these inputs, agricultural productivity is still at its lowest level, compounded by weak institutions, imperfections in the credit market, poor infrastructure, and the lack of advanced research and advisory systems [12]. The contribution of CSA to food security and agricultural income by increasing agricultural productivity is derived from the literature [13, 14]. Therefore, it makes sense to thoroughly examine the productivity impacts of climate-smart agricultural practices that help policymakers scale up these practices and to understand the magnitude and potential of their impacts in improving the welfare of smallholders. Therefore, this study focused on examining the impact of climate-smart agriculture practices (e.g., row planting) on productivity.

To the best of our knowledge, there are few studies on the effects of row planting on household welfare (wheat productivity per land, in our case). Tolosa et al. [6] studied the effect of row planting of wheat on yield in the Arsi Zone, Oromia region. Their study concludes that the effects of row planting are related to agroecology, where row planting is significant in upland areas and insignificant in lowland areas. Tamirat & Abafita [15] analyzed the effects of row planting on yield, farm income, and household expenditure among wheat farmers in the Duna district, SNNP region. Accordingly, their result evidenced that the introduction of row planting is associated with higher yield and higher household expenses. Mengie et al. [8] studied the effects of sowing and sowing rate on teff yield and yield components in Adet, Northwest Ethiopia. In their study, a 5 kg/ha seed rate and row sowing result in higher yields and maximum net returns for teff growers in the region. Fentie & Beyene [5] studied the acceptability and effects of row planting on the welfare (farm income and household expenditure per capita) of rural teff producing farmers in the Gubalafto district, Amhara region. They found that row-planting adopters had higher per capita consumption and income per hectare than nonadopters. Habtewold [16] also used a 2015 Ethiopian socioeconomic survey (ESS Wave3) to examine the country-level impacts of row planting and fertilizer use on the multidimensional poverty of rural households in Ethiopia. He found that introducing row planting and chemical fertilizers together was significantly associated with poverty reduction. In contrast to these studies, Vandercasteelen et al. [17] examined the effects of teff row planting in Ethiopia and that row planting is associated with increased labor demand and reduced labor productivity. They also showed that planting in rows had no significant correlation with yield.

Furthermore, the evidence on the superiority of row planting over traditional broadcasting methods is mainly qualitative and based on agronomic knowledge [6]; Abraha et al. [18] and Tamirat and Abafita [15]. Studies on the impacts of row planting on the welfare of rural smallholder farmers are scant in the empirical literature, and existing studies lack agreement on the impact of row planting. Besides this, available studies applied different methodologies and measured smallholder welfare by crop yield.

Against this background, our study aims to assess the impact of row planting on wheat productivity of rural smallholder households in the district of Misha, South Nation, and Nationalities Region, and the contribution of our study is manifold. First, we measure smallholder farmers' welfare in productivity (wheat yield/ha). Second, we applied Propensity Score Matching (PSM) with the generalized Roy model's semiparametric Local Instrumental Variable (LIV) method. The Roy model is superior to the linear endogenous treatment effects and maximum likelihood estimation of switching regression because the Roy-LIV model estimates the marginal treatment effect (MTE) and the average treatment effect (ATE) of the adoption decision [19]. In addition, the Roy model accounts for the selection of unobservable and the returns (outcome variable) (“The problem of selection on returns happens when the adoption decision of row planting and unobserved variables are essentially related (essential heterogeneity) that affects the return of the adoption (outcome variables), and it is this dependence that makes it relevant to examine the marginal effect of adoption of the row planting” [5].). Third, our study complements the scant literature on the relationship between adopting climate-smart agricultural practices and the welfare of smallholder households in rural areas. Fourth, it helps put the issue on the policy agenda by quantifying the impacts of row planting practices on productivity. Finally, our study closes the knowledge gap by introducing climate-smart agricultural practices on board.

Therefore, the major objective of this study is to investigate the impact of climate-smart agriculture practices on rural household welfare in the southern region of Ethiopia. First, our study investigated the quantitative impact of wheat row planting on the productivity of smallholder farmers. Second, the study examined the marginal treatment effect of row planting adoption. Based on these objectives, our study forwarded the following hypothesis.

- hypothesis 1: Row-planting technique enhances the productivity of smallholder farmers
- hypothesis 2: Row-planting adoption increases the marginal utility of farmers to adapt climate-smart agriculture practices.

We organized the rest of our work as follows.  Section 2 dedicates to the overview of wheat production and row planting adoption; Section 3 deals with the data sources, sampling, and study area description.  Section 4 covers the empirical strategy, Section 5 contains the findings and discussions, and Section 6 highlights the conclusions and policy implications of adopting row planting.

## 2. Overview of Wheat Production and Row Planting in Ethiopia

Wheat is an important food crop grown in the highland areas of Ethiopia. It plays an essential role in maintaining food security and poverty alleviation in Ethiopia [20]. In addition to its essential contribution to the food system, it plays a prominent role as its straw is used as animal feed, as a house roof, and as a cohesive material for building houses. It is the third most cultivated crop in terms of acreage and proportion of total crop production, after teff and maize [21]. It is also the second crop marketed internationally [22], and its contribution to food security is of strategic importance [23].

Wheat is a strategic staple in Africa, and its demand increases over time due to income growth and urbanization. However, sub-Saharan countries produce around 30% of their domestic needs, meaning these countries are heavily dependent on imports. Dependence on imports carries the risk of commodity price volatility and supply shocks [24]. In Ethiopia, over 4.7 million farmers grow wheat, and about 13% of those farmers are from the South Nation and Nationalities (SNNP) region. Despite its lower national production share, the region's wheat productivity is the second highest (26.67 quintals/ha) after the Oromia region (29.71 quintals/ha) [25]. Although wheat is grown as a grain, production is insufficient to meet domestic wheat consumption demand, meaning Ethiopia remains a net importer of wheat. It covers 25% of domestic needs through imports. The lower wheat production is mainly due to traditional production techniques dominated by small farmers and dependence on rain-fed agriculture. One of the traditional production systems is the seed sowing by broadcast. Sowing through broadcast reduces yield, as it requires a higher amount of seed, resulting in less plant space and thus reduces plant nutrients. A smaller plant space also makes weeding more difficult [7].

To increase productivity, climate-friendly farming practices have recently been introduced, including row plantings. This production technology has several advantages that we cannot achieve with the traditional broadcast method. For example, planting in rows helps increase the spacing between plants, allowing better access to water and sunlight and reducing the amount of seed.

## 3. Materials and Methods

This study was conducted in the Misha district of southern Ethiopia using household-level survey data collected through a structured questionnaire. According to the population projection of the Central Statistical Office, the Mischa district had 170,490 inhabitants in July 2021; of that, 48.6 per cent were men and 51.4 per cent were women. Around 95 per cent of the population lives in rural areas and depends on agriculture. The Misha District has three agro-climatic zones: lowland (Kola, 10%), mid-altitude (Woina Dega, 70%), and highland (Dega, 20%) [26]. The most common crops are wheat, teff, corn, sorghum, peas, and beans. In addition, the district also grows cash crops such as chat, coffee, and vegetables.

We used a multistage sampling method to select the study participants. For this study, we first purposively chose Misha Woreda because wheat is the dominant crop in the area. Second, we selected three kebele (Kebele is the smallest administrative unit), namely Gidasha, Ololico, and Forks kebele, that primarily produce wheat. Third, we randomly select 202 households that produce improved wheat from the three kebele. Of that, 83 households are adopters and 119 households are nonadopters of row planting technology in their wheat farm. The sample size was determined based on the proportion to the size of the household in each kebele. In the first step, we selected those farmers who produce improved wheat varieties in the study area. In the second phase, we divided these farmers into producers of an improved wheat variety using row seeding technology and traditional broad seeding technology. Finally, we randomly selected wheat producers from each treatment status category in the kebele sample and collected the data through a structured questionnaire in the 2018/19 cropping season.

## 4. Empirical Strategy

The main challenge in evaluating an intervention or program is obtaining a credible counterfactual estimate: What would have happened to the participating units if they had not participated? [5]. Therefore, identifying the counterfactual problem is a proper impact assessment [27]. If the treatment is randomly assigned, the outcome of interest in no treatment scenario is a reasonable estimate of the counterfactual outcome. However, treated households may have different characteristics than untreated households. Farmers are free to choose, and decisions are likely to be influenced by unobserved human (motivation, innovation) and agricultural (fertility) traits and experimental factors that can be correlated with the outcome variables [9, 28] and [10]. As a result, the adoption decision is potentially endogenous, and thus, comparing the row planting technology outcomes with the welfare of the two groups using the OLS technique leads to biased estimates [29].

Based on Heckman et al. [30], one can estimate the effects of row planting on the mean outcome of the treatment group (adopters) and the mean outcome of the control group (nonadopters) as follows: Π = *Y*_1_–Y_0_. This is the difference between the outcome of the treatment group and the controlled groups. However, although it is mathematically simple, estimating impacts using the above equation leads to a “lack of data problem” referred to in the program evaluation literature [31]. One primary reason is that the outcomes of the treatment and control groups cannot be observed simultaneously for a single person. Therefore, with the nonrandom assignment of samples to participation, a simple difference in mean outcome between the treatment and control groups cannot predict the mean treatment effect of row planting technology [30]. Therefore, it is essential to find a valid counterfactual to assess the treatment effects in nonrandom experiments.

Thus, for our study, the average treatment effect on treated (ATT) is given by the following:

(1) $$\begin{array}{c} \prod_{\text{ATT}}=\text{ATT}\left(\partial X_{i};Z_{i}=1\right)=E\left(Y_{1}-Y_{0}\right)=E\left(Y_{1},Z_{i}=1\right)-E\left(Y_{0},Z_{i}=1\right), \end{array}$$

where *Z*_i_ is the binary treatment variable and indicates the treatment status of the household *i;* it takes on the value one of the households adopted row planting and zero, otherwise (adopted broadcasting method), and *X*_i_ is the set of controlled covariates. *E* (Y_1_, *Z*_i_ = 1) refers to the mean outcome for the treatment group, and *E* (Y_0_, *Z*_i_ = 1) refers to the mean outcome of the controlled group. While the mean outcome for the treated group is observable, the mean outcome for the untreated group is not. Therefore, we need to find groups that share characteristics similar to the treatment group to arrive at an accurate estimate of the average treatment effect (ATT). To this end, we used a propensity score matching method to find a controlled group that matched with the treatment group and to estimate the average treatment effect of adopting wheat row plantings. In the empirical literature, there have been different approaches to estimating the treatment effects. Other approaches, such as the endogenous switching regression model [32–36] and endogenous treatment regression model [33, 37], can also potentially be used to estimate the treatment effects of row planting adoption on farm productivity. However, these approaches usually require at least one valid instrumental variable and it is often hard to identify a valid instrumental variable. Therefore, this study employs propensity score matching that does not rely on an instrumental variable for estimations. The use of propensity score matching is also common in the empirical literature [38–41].

A covariate balancing test is required to control the bias and see if it changes after matching, making the matching group an appropriate counterfactual for the treated group. However, there are different versions of balance tests in the literature [31]. Accordingly, we examine whether the treatment effects are sensitive to hidden bias by following the procedure of Rosenbaum & Rubin [42] by testing the standardized differences in the means of each variable in *Z* between the treatment and matched comparison group samples. If the standardized bias difference is greater than 20%, it signals a failure of the match. However, our results are insensitive to hidden biases.

A major problem with propensity score matching is that it fails to account for unobserved heterogeneity. Therefore, we fitted the generalized Roy model with local instrumental variables to complement the propensity score matching results. As a result, the Roy model underpins the selection of unobservable factors, estimates the marginal treatment effect, and the mean treatment effect of the decision to enter treatment using parametric and nonparametric approaches [19].

## 5. Results and Discussion

### 5.1. Descriptive Statistics

After a thorough analysis of the related empirical literature, we selected the variables presented in Table 1. Table 1 shows that 41.1 per cent of the sample participants adopted the row planting method for improved wheat production. In addition, men head about 52 per cent of adopter households, while only 41 per cent of nonadopters are male-headed households. The gender composition of adopters and nonadopters is statistically significant, as shown in Table 1. While most demographic variables show a significant mean difference, institutional variables show no significant difference between the treatment and control groups. As shown in Table 1; however, there is a significant difference in the means of gender and market information (at the 10% significance level), social role, off-farm income (at the 5% significance level), and cooperative membership (at the 1% significance level) between users and nonusers of the row planting method of agricultural production.

Compared to nonadopters of row planting technology, adopters are male, have larger household sizes, are younger, are closer to advisory offices, are cooperative members, have more social roles, and are involved in nonfarm activities. The average household size of adopters is 4.5 people compared to 4.4 people for nonadopters, although the difference is statistically insignificant. The choice of wheat row planting is positively associated with access to market information, membership in cooperatives, social role, and off-farm income. Access to these institutional services could help farmers adapt yield-enhancing technologies. Because, on the one hand, farmers have the opportunity to observe, learn, and develop knowledge about the use and application of improved technologies, and on the other hand, farmers become risk-takers. Adopters also consume more fertilizers and have larger farm sizes compared to nonadopters.

The other important variable is the outcome variable, that is, wheat productivity as measured by yield per hectare of land. Adopters have higher wheat productivity than nonadopters. There is a significant difference in the average productivity of wheat production between users and nonusers of the row sowing method. Adopters produce about 21 quintals (2124.696 kg/ha) per hectare, and nonadopters produce eight quintals (800.489 kg/ha) per hectare of land, which is significant at the 1 per cent significance level.

Finally, we asked respondents about their reasons for adopting row planting technology in their production. Approximately 90% of adopter respondents cited improving productivity, reducing seed requirement (70%), and easing weeding (40%) as the first, second, and third reasons for adopting row planting. Similarly, we asked the nonadopters why they did not apply the row-planting method in their wheat production. Accordingly, about 85% of them reported that the technology is labor intensive and time-consuming. The result prompts the need to use complementary technologies, such as agricultural mechanization, that reduce labor and time requirements, thereby increasing nonuser's acceptance of row planting technology.

### 5.2. Determinants of Adoption, Propensity Score, and Matching

This section highlights the estimation of propensity scores and matching based on the propensity score. After calculating the propensity score, we produced the matched controls with the corresponding treatment samples. Then we assessed the average treatment effects on persons treated (ATT) based on the matched households.

### 5.3. Determinants of Row Planting Adoption

Table 2 shows the marginal effect estimates of the introduction of wheat row planting technology. As the table shows, household size, a proxy variable for labor requirement, is unrelated to household adoption decisions. However, the coefficient is positive, suggesting some positive association between household size and adoption. The result is consistent with the findings of [5, 43]. The result has implications for reducing nonadopters labour restrictions, leading them to adopt row planting via using hired labor. Demographic variables such as education, gender, and household head age have a positive and significant impact on the household's adoption decisions. The results of the selection model showed that one year more education of the household head leads to a four-percentage point increase in the likelihood of adoption. Educated farming households are more likely to adapt to improved technology than their counterparts because they can process information quickly, learn how to use it, and easily develop the skills needed to apply new technologies. Our result confirms the results of the studies in [44] and [45].

The outcome of the selection model also shows that a male householder is positively associated with the adoption of row planting technology. Male-headed households are 31.7 per cent more likely to adapt than their female counterparts are. The result is similar to the results of Admassie & Ayele [46], who argued that male-headed households are more likely to adapt to new technologies because they are more resourced and have access to new information and ideas. Ragasa et al. [47] also argued that female households are less likely to receive extension services and even if they do receive, the quality of the services can be compromised.

Age is another variable positively associated with household adoption decisions, but only limited. The effect of the age of the household head on technology adoption was not clear in the literature [45, 46]. On the one hand, older households have more farming experience, resources, and responsibilities to make decisions and are more likely to adapt innovative technologies. On the other hand, older farmers may be untrained, less exposed to information, risk-averse, and happily ignoring new technology. Considering this inconclusive result, in the empirical studies, we included the squared age in the selection model to see if age had a nonlinear relationship with the probability of farmer adoption. Our result reveals a nonlinear relationship between the age of the head of the household and the decision to adapt. The age of farmers is a measure of the farmer's agricultural experience that increases the likelihood of adopting new technologies but only up to a certain age limit. After a certain age, households become less proactive and stick to using the technology they adopted when they were younger. Therefore, they prefer to avoid risks related to the use of new technologies. As described in Table 2 below, an additional year of farmers increases their chances of adoption by 5.4%, while this reverses with older age as the coefficient of squared age is negative and significant at the 5% level of significance. Therefore, we conclude that age and adoption do not have a clear relationship that warrants further analysis using a large dataset.

Among the institutional variables, membership in cooperatives is positively associated with row planting technology. Farmer's membership in cooperatives significantly increases the probability of adoption by 26.9 percentage points. Because cooperatives serve as a source of information, provide inputs and offer training of various kinds to enable farmers to develop new skills in using new technologies and become more risk-takers. The role of cooperative membership in promoting agricultural technology adoption has been widely proved in the literature, and our findings are in line with the findings of [37, 48, 49]. Wossen et al. [48] argued that cooperative membership of households increases their asset holdings and the likelihood of formal credit access. Thus, membership in cooperative is strongly and positively associated with the adoption of new technologies. Zhang et al. [49] also found that membership in agricultural cooperatives increase the likelihood of farmers in adopting extensive agricultural technologies. Agricultural cooperatives help to accelerate technological progress and its spread among farmers, as cooperatives promote new technologies by organizing training and initiating cooperation with research and development institutions.

### 5.4. Propensity Score and Matching

After estimating the logit model using the covariates in Table 2, we use these variables and predict the propensity score to find a matched sample of households. Accordingly, the predicted propensity score for adopter farmers is within a bound of [0.212 and 0.903] with a mean of 0.469. The propensity score for nonadopters ranges from 0.135 to 0.817, with a mean of 0.367. Thus, the common support condition is met in the range of 0.212 to 0.903.

**Figure 1:**
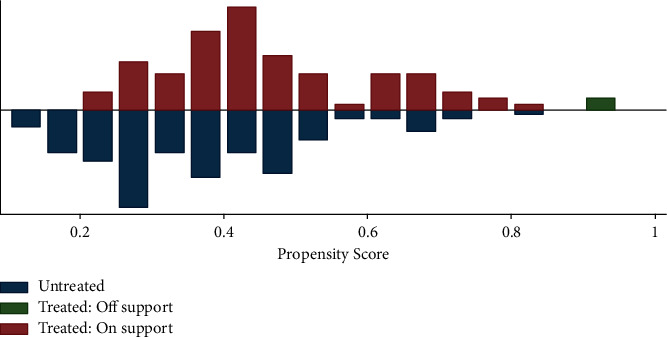
Distribution of propensity score (80 of 83 treated samples are in the common support region).

We plot the common region of support that balances household characteristics between adopters and nonadopters of row planting technology using the predicted propensity score. The presence of a significant number of samples of the two groups in the common support region indicates the presence of an appropriate balance in the distribution of covariates between the treated and controlled samples. When covariate imbalances occur, it is just accidental. To check the fulfillment of the balancing condition, we introduced the common support condition in the estimation by matching the region of common support.  Figure 1 is a visual representation of the overall support condition, showing that respondents share common support or overlap in the distribution of treated and untreated propensity scores. In addition to visualizing the balance test, we performed a standardized bias test and found that the mean bias is well below 20%, the pseudo R2 is 0.031, and the *p*-value of LR (*χ2*) is 0.935. Thus, the distribution of covariates between the treated and control groups is balanced after matching.

### 5.5. Estimation of Average Treatment Effect on Treated (ATT)

Using four comparative techniques, we estimated the effects of adopting row-planting technology on the welfare of farmers' growing improved wheat. These techniques are the nearest neighbor (NN) matching, radius matching, kernel matching, and stratification matching algorithms. Estimations are using Bootstrap standard errors.

The estimates of all matching techniques showed the positive and significant effect of row planting on wheat productivity for the treated groups. As shown in Table 3 below, the average treatment effect on treated (ATT) ranges from 1299 kg to 1368 kg per hectare of land, which means that the increase in wheat productivity ranges from 12.99 quintals to 13.68 quintals per hectare for the row planters compared to the broadcasters. Our results agree with Tolosa et al. [6], who found that applying the row planting method increased wheat yield by 14% more than applying the broadcasting method in the highland area of Ethiopia. Tamirat and Abafita [15] also found that row planting increased the yield of wheat farmers by almost 75% and 7.23 quintals per hectare more than that of untreated wheat farmers. Similarly, our result is consistent with the results of Fentie and Beyene [5], who found that the per capita consumption of households growing teff using the row planting method increased by 12.3% to 18.4% compared to those using the broadcasting method. However, our result contrasts the results of Vandercasteelen et al. [17], who found that planting in rows has no significant impact on teff yield. Their results also confirmed that planting in rows is associated with an increased workload.

Despite the difference in the size of the average treatment effect from using all matching algorithms, row planting has a positive effect that shows its essential role in increasing wheat productivity for the treated farmers. Therefore, our result has implications for broadcasters that should produce wheat by sowing in a row instead of using the traditional broadcast method.

### 5.6. Sensitivity Analysis

Sensitivity analysis verifies the sensitivity of the estimated treatment effect to small deviations in the model's specification, which is a strong assumption that must be maintained to have the pure effect of interventions on outcome variables. In the empirical literature, Rosenbaum & Rubin [42] recommend bounding methods to test whether estimates of mean treatment effects on those treated (ATT) are responsive to external changes other than treatment. Accordingly, the sensitivity analysis presented in Table 4 below confirms the absence of hidden biases and confirms that the estimated treatment effects for wheat farmers are solely due to the introduction of row planting technology. This result is similar to studies by [5, 15] analyzing the effects of row planting technology.

### 5.7. Semiparametric Local Instrumental Variable (LIV) Model Estimation Results

**Figure 2:**
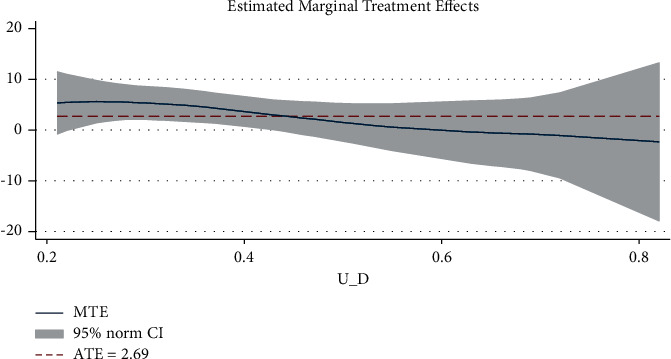
Marginal Treatment Effect over the Common Support of p (Z).

The results of semiparametric LIV are similar to those of propensity score matching with positive mean treatment effects. The marginal treatment effect (MTE) increases with a higher probability of participating in the treatment.  Figure 2 is the graph of the marginal treatment effect result showing that the marginal treatment effect is a negative function of propensity to adapt (UT). Therefore, the marginal utility of using row-planting technology increases as farmers' propensity to use row planting technology increases.

**Figure 3:**
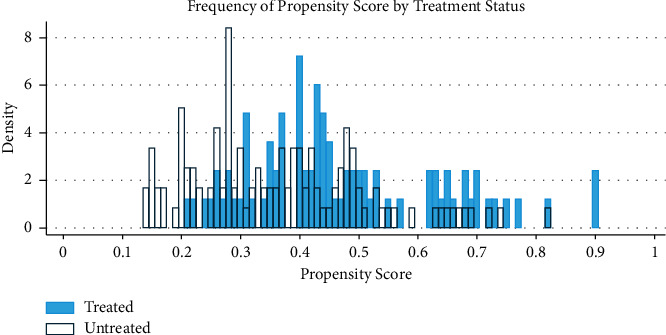
The common support of P (Z).

The MTE is estimated using the semiparametric LIV method with values within the common support of the predicted propensity scores. Accordingly, as shown in Figure 3 below, the MTE showed the tendency toward treatment was higher in the row planters (treated group) than in the untreated group. The result of the LIV semiparametric regression is shown in Table 5, and the last row of the table is the mean treatment effect (ATE), which is positive and statistically significant. Thus, the marginal treatment effect of producing wheat using the row seeding method increases the productivity of wheat-growing households.

## 6. Conclusions and Policy Implications of the Study

The empirical evidence shows that the relationship between climate-smart agricultural technologies and their impact on well-being is unexplored, and the available evidence is mixed. It is challenging to find appropriate methods to analyze the quantitative impacts of climate-friendly practices, and therefore, there are few studies on the uptake of climate-smart agriculture technologies and their impact on the productivity of rural farm households. Therefore, this study examined the effects of CSA (row planting) technology on wheat-producing farm households' welfare (measured in terms of productivity) using state-of-the-art analysis methods. We used propensity score matching and the semiparametric LIV method to estimate the mean treatment effect of introducing row plantings to treated sample households. In addition, the sensitivity analysis of the estimated treatment effects using the Rosenbaum limit method is verified, and the ATT estimates are the pure effects of applying the row planting technique.

The results of our study showed the significant and positive contribution of row planting technology to the productivity of wheat-producing farmers. Propensity Score Matching created appropriate matching samples for the treatment groups, and the estimates confirm that the average productivity of the row seeders is higher than that of the broadcasters. Furthermore, as confirmed by the sensitivity analysis, the treatment effect is free from hidden bias. Thus, for adopter households, the increase in productivity is associated only with sowing wheat in rows. We also estimated the marginal treatment effect of introducing row plantings using the semi-parametric LIV model. The results showed that the marginal benefit of adopting row-planting increases as the likelihood of farmers adapting climate-smart agriculture practices.

Our study also explained why farmers use row planting technology for those who adapt it and why they do not use it for nonusers. Accordingly, a more significant proportion of adopters responded that the need for productivity and ease of weeding are the main reasons for adopting the technology. On the other hand, nonadopters reported that labor and time limitations are the challenges of adopting the technology. Therefore, efforts to disseminate this technology should focus on helping farmers with labor allocation and time management or helping them by providing agricultural mechanization (reducing the need for labor).

Our result showed that education, gender, age, and cooperative membership are strongly associated with row planting adoption. The main implication is that the positive impact of introducing row planting on farmer productivity could be enhanced by encouraging farmer membership in cooperatives, supporting female-headed households with farm information and access to formal credit, and increasing access to education.

The results of our study correspond to similar studies on the introduction of agricultural technology. Further expansion of the technology to other but similar geographic features could increase farmer acceptance of the technology and improve farm household well-being by increasing productivity. Aside from these, farmers exposure to climate change could also be less since the technology is climate-friendly. However, we encourage other researchers interested in this area to analyze large datasets across different geographical regions to generalize the results on a larger scale.

## Figures and Tables

**Table 1:** Balance test of covariates.

Variable	Adopters (*n* = 83	Non-adopters (*n* = 119)	Mean difference (non- adopters—Adopters)
Household size	4.506 (1.81)	4.437 (2.208)	−0.69 (0.293)
Education of household head (number of years of formal schooling)	2.711 (3.319)	2.924 (3.128)	0.214 (0.458)
Age of the household head	42.651 (9.863)	42.664 (11.06)	0.013 (1.513)
Livestock(TLU)	1.627 (1.009)	1.723 (1.186)	0 .096 (0.160)
Extension (distance from extension offices in walking minutes)	25.422 (14.164)	25.563 (13.087)	0 .141 (1.936)
Gender (1 if the head is male)	0.518 (0.503)	0.412 (0.494)	−0.106^*∗*^ (0.712)
Spouse education (1 if spouse can read and write)	0.048 (0.215)	0.05 (.22)	0.002 (0.312)
Farm size (area cultivated for wheat production)	2.027 (0.913)	1.981 (0.883)	−0.046 (0.128)
Cooperative membership (1 if the household head is member of farm cooperative)	0.313 (0.467)	0.126 (0.333)	−0.187^*∗∗∗*^ (0.056)
Off-farm (1 if the household has off-farm income source)	0.12 (0.328)	0.042 (0.201)	−0.078^*∗∗*^ (0.037)
Social role (1 if the household head has any role in the community)	0.217 (0.415)	0.118 (0.324)	−0.099^*∗∗*^ (0.052)
Market information	0.289 (0.456)	0.202 (0.403)	−0.087^*∗*^ (0.061)
Farm experience (years of farm experience)	15.795 (8.606)	16.008 (8.984)	0.213 (1.263)
Mkt distance (distance to the main market in walking minutes)	37.289 (12.393)	37.202 (11.34)	−0.087 (1.685)
Access to credit	0.313 (0.467)	0.311 (0.465)	−0.002 (0.066)
Fertilizer use	0.289 (0.020)	0.143 (0.032)	0.146^*∗∗∗*^ (0.057)

Numbers in parenthesis are standard errors and ^*∗*^, ^*∗∗*^, and ^*∗∗∗*^ are significance levels at 10%, 5%, and 1% level of significance

**Table 2:** Marginal effect estimation of the Logit model.

Variables	Marginal effect	*p*-value
Household size	0.005	0.756
Education of household head	0.040	0.045^*∗∗*^
Sex of the household head	0.317	0.009^*∗∗∗*^
Age of the household head	0.054	0.036^*∗∗*^
Age squared of the household head	−0.001	0.042^*∗∗*^
Livestock ownership (TLU)	−0.045	0.141
Distance from extension offices	−0.021	0.222
Farm size (ha)	0.026	0.501
Fertilizer use	0.065	0.515
Cooperative membership	0.269	0.012^*∗∗*^
Distance from the main market	0.026	0.179
Access to credit	−0.111	0.162
Access to market information	0.059	0.482

^*∗∗*^, and ^*∗∗∗*^ refers to significance at 5% and 1% level of significance.

**Table 3:** Estimation of average treatment effect (ATT): estimating the impact of row planting on wheat productivity.

	NN-matching	Radius(0.1)	Kernel	Stratification
ATT	1368.742^*∗∗∗*^	1299.519^*∗∗∗*^	1330.209^*∗∗∗*^	1348.326^*∗∗∗*^
SE	293.548	341.583	276.064	387.914
Treated	83	83	83	83
Control	53	100	100	100

^*∗∗∗*^ implies significance at a 1% level of significance.

**Table 4:** Results of Sensitivity analysis using Rosenbaum bounds for wheat productivity. Rosenbaum bounds for Productivity (*N* = 81 matched pairs).

Gamma	Sig + upper bound significance level	Sig-lower bound significance level	t-hat + upper bound Hodges-Lehmann point estimate	t-hat-lower bound estimate Hodges-Lehmann point estimate	CI + upper bound confidence interval (a = .95)	CI-lower bound confidence interval (a = .95)
1	0	0	889.238	889.238	533.333	1287.69
1.25	0	0	726.926	1057.11	384	1485
1.5	0	0	603.167	1209.24	268.667	1638.39
1.75	0	0	500.667	1320.25	179.417	1795.9
2	0	0	418.667	1433.23	92.9167	1957.67

^*∗*^Gamma refers to odds of differential assignment due to observed factors.

**Table 5:** Determinants and impact of row planting semiparametric LIV regression.

Parameters	Estimates
Household size	−0.267^*∗∗∗*^ (0.008)
Education	−0.276^*∗*^ (0.076)
Gender	−1.334 (0.109)
Age	0.022 (0.728)
Farm experience	−0.026 (0.714)
Livestock ownership(TLU)	0.107 (0.589)
Extension	−0.041 (0.196)
Farm size	−0.423 (0.101)
Fertilizer use	−0.585 (0.638)
Off-farm activities	−1.691 (0.389)
Access to credit	−0.178 (0.732)
hfsXp (interaction of household size and pscore)	0.631^*∗∗∗*^ (0.004)
educXp (interaction of education and P score)	0.374 (0.343)
genXp (interaction of gender and pscore)	1.567 (0.452)
ageXp (interaction of age and pscore)	0.048 (0.756)
farm_expXp (interaction of farm experience and pscore)	0.052 (0.787)
livestockXp (interaction of livestock ownership and pscore)	−0.254 (0.618)
ExtensionXp (interaction of extension visit and pscore)	0.004 (0.954)
Farm-sizeXp (interaction of farm-size and pscore)	0.961 (0.106)
fertilizerXp (interaction of fertilizer use and pscore)	0.987 (0.680)
off_farmXp (interaction of off-farm participation and pscore)	2.787 (0.353)
creditXp (interaction of access to credit and P score)	0.580 (0.651)
E Y1–Y0)@X	2.687^*∗∗*^ (0.026)

Note that the outcome variable is the log of productivity. ^*∗∗∗*^ significant at a 1% level of significance. ^*∗∗*^ significant at a 5% level of significance. ^*∗*^ significant at a 10% level of significance.

## Data Availability

The data that support the findings of this study are available from the author upon request.
